# Unmasking inequalities: Sub-national maternal and child mortality data from two urban slums in Lagos, Nigeria tells the story

**DOI:** 10.1371/journal.pone.0177190

**Published:** 2017-05-10

**Authors:** Erin Anastasi, Ekanem Ekanem, Olivia Hill, Agnes Adebayo Oluwakemi, Oluwatosin Abayomi, Andrea Bernasconi

**Affiliations:** 1 United Nations Population Fund, New York, United States of America; 2 Operational Centre Barcelona, Médecins sans Frontières / Doctors without Borders, Barcelona, Spain; 3 Department of Community Health & Primary Care, College of Medicine, University of Lagos, Lagos, Nigeria; 4 Turquoise Zeta Consult, Lagos, Nigeria; 5 Swiss Tropical and Public Health Institute, Basel, Switzerland; 6 University of Basel, Basel, Switzerland; Massachusetts General Hospital, UNITED STATES

## Abstract

**Introduction:**

Nigeria has one of the highest maternal mortality ratios in the world as well as high perinatal mortality. Unfortunately, the country does not have the resources to assess this critical indicator with the conventional health information system and measuring its progress toward the goal of ending preventable maternal deaths is almost impossible. Médecins Sans Frontières (MSF) conducted a cross-sectional study to assess maternal and perinatal mortality in Makoko Riverine and Badia East, two of the most vulnerable slums of Lagos.

**Materials and methods:**

The study was a cross-sectional, community-based household survey. Nearly 4,000 households were surveyed. The sisterhood method was utilized to estimate maternal mortality and the preceding births technique was used to estimate newborn and child mortality. Questions regarding health seeking behavior were posed to female interviewees and self-reported data were collected.

**Results:**

Data was collected from 3963 respondents for a total of 7018 sisters ever married. The maternal mortality ratio was calculated at 1,050/100,000 live births (95% CI: 894–1215), and the lifetime risk of maternal death at 1:18. The neonatal mortality rate was extracted from 1967 pregnancies reported and was estimated at 28.4/1,000; infant mortality at 43.8/1,000 and under-five mortality at 103/1,000. Living in Badia, giving birth at home and belonging to the Egun ethnic group were associated with higher perinatal mortality. Half of the last pregnancies were reportedly delivered in private health facilities. Proximity to home was the main influencing factor (32.4%) associated with delivery at the health facility.

**Discussion:**

The maternal mortality ratio found in these urban slum populations within Lagos is extremely high, compared to the figure estimated for Lagos State of 545 per 100,000 live births. Urgent attention is required to address these neglected and vulnerable neighborhoods. Efforts should be invested in obtaining data from poor, marginalized, and hard-to-reach populations in order to identify pockets of marginalization needing additional resources and tailored approaches to guarantee equitable treatment and timely access to quality health services for vulnerable groups. This study demonstrates the importance of sub-regional, disaggregated data to identify and redress inequities that exist among poor, remote, vulnerable populations—as in the urban slums of Lagos.

## Introduction

Between 1990 and 2015, maternal mortality, defined as the number of women who die from pregnancy-related causes while pregnant or within 42 days of pregnancy termination per 100,000 live births, dropped by about 44% globally, yet, still every year, almost 300,000 women die from pregnancy and childbirth related complications [1]. The high number of maternal deaths in some areas of the world reflects inequities in access to quality health services, and highlights the gap between rich and poor, rural and urban settings, resulting, in 2015, in a maternal mortality ratio in low-income countries of 239 per 100,000 live births, as compared to 12 per 100,000 live births in developed countries.

Nigeria is still struggling to reduce maternal mortality and, unfortunately, failed to meet the Millenium Development Goals (MDG) target. In Nigeria, the maternal mortality ratio (MMR) has dropped from 1350/100,000 live births in 1990 to 867/100,000 live births (95% CI: 673–1130) in 2010 and finally to 814/100,000 live births (95% CI: 596–1180) in 2015 [2]. In Lagos State, MMR is estimated at 545/100,000 [3]. A woman’s chance of dying from pregnancy and childbirth in Nigeria is estimated at 1 in 13. These figures vary widely between regions within the country, with rural settings typically suffering higher mortality than urban areas [3, 4, 5, 6]. Although many of these deaths are preventable, the coverage and quality of health care services in Nigeria continue to fail too many women and children, especially the most vulnerable and marginalized. Presently, less than 20% of health facilities offer emergency obstetric care and only 35% of deliveries are attended by skilled birth attendants [7, 8].

These figures may even be underestimated due to imperfect data collection. In Nigeria there is a passive system of registering all births and deaths, but this system is grossly inadequate for estimating MMR. Because of this inadequacy, most MMR data reported in the literature are hospital-based. Therefore maternal deaths which occur at home or with a traditional birth attendant (TBA) are not captured in reported hospital data. Another source of data is the Nigerian Demographic and Health Survey (NDHS) which is conducted every 5 years and which generates national estimates on a number of key demographic and health indices.

When the survival of the mother during pregnancy and childbirth is at risk, the life of the newborn is also threatened. The perinatal mortality rate (PMR) is still high in Nigeria. PMR is estimated to account for more than 20% of the total deaths before the age of 5, largely due to obstetrical complications that could be prevented or remedied by timely, proper, quality antenatal, intrapartum, and postpartum care for mother and baby, including access to skilled birth attendance and emergency obstetric and newborn care, as needed [9]. This important key indicator of the health status of the population, is estimated at 76 per 1000 births (i.e., out of 5.3 million babies born in 2004, there were an estimated 425 000 perinatal deaths) [10]. Like MMR, PMR also differs from region to region with higher mortality typically found in the Northern Regions [11, 12, 13, 14], reflecting the inequalities within the country.

Médecins Sans Frontières Operational Centre Barcelona (MSF-OCBA) began offering health services and treating patients in Lagos from 2009 to 2012. MSF supported the Outpatient Department (OPD) and Inpatient Department (IPD) facility in Aiyetoro Hospital and operated two mobile clinics for the most vulnerable areas. Two neighborhoods were of particular, critical interest to MSF: the areas of Makoko Riverine (Yaba Local Council Development Area) and Badia East (Apapa Local Government Area). These areas represent the highest levels of urban blight. The population in Makoko Riverine is estimated at approximately 40,000, mainly emigrated from Benin Republic many decades ago due to economic reasons and family reunification. They are typically French- or Egun-speaking, living in a floating settlement, isolated from the rest of the city and, thus, relatively neglected [15].

The community of Badia East (estimated at approximately 20,000 inhabitants) is characterized by a large presence of sex workers (SW) and an alarming situation of urban violence. The populations of these two slums have difficulties accessing health care services due to perceived discrimination, among other barriers.

## Materials and methods

We conducted a cross-sectional, community-based household survey, with the primary objective of estimating the maternal mortality ratio through the direct sisterhood method [16]. We also aimed to estimate perinatal mortality and to assess women’s health seeking behavior around pregnancy and childbirth. Perinatal mortality was assessed through the preceding births technique [17, 18, 19]. Data were collected from February to March 2012 within the framework of the ongoing activities of MSF-OCBA in Lagos. Ethical approval for this study was obtained from Lagos University Teaching Hospital, the Lagos State Ministry of Health and MSF’s Ethical Review Board.

The survey targeted all males and females living in the urban slum communities of Makoko Riverine and Badia East between the ages of 15 and 49 years, who voluntarily agreed to participate in the study.

The sampling frame was the population of each of the 2 catchment communities (Badia East and Makoko Riverine) and the sampling unit was the household. Data were obtained from approximately 4,000 households, randomly selected in two stages. Following a probability proportional to size approach, systematic random sampling was used to select households within the study communities. By proportion, as originally planned, 2,666 households (66.5%) were to be randomly selected in Makoko Riverine and 1,334 (33.5%) in Badia East, for an overall total of 4,000 households. However, after the study began, these proportions were adjusted slightly–increased for Makoko Riverine (to 3,015) and decreased for Badia East (to 948)–to take into account the unfortunate and unforeseen demolition of houses and consequent displacement of persons that occurred during the survey in Badia East, as well as the likely initial overestimation of the population residing full-time in Badia, as confirmed by MSF community outreach workers, in consultation with local community leaders in Badia East.

Since estimating the maternal mortality ratio was the primary objective of this study, by estimating a total fertility rate (TFR) of 5.4, this sample size was chosen to be large enough to detect a MMR of 500 with a margin of error of 20% and a confidence interval of 95% [20].

One female or male who met the inclusion criteria of the study was randomly selected per household for the interview. In cases where no eligible respondent was present in the household, data collectors marked the house for a return visit. If, after one further attempt, still no eligible respondent was found at home, data collectors went on to the next nearest household with an eligible respondent present.

In case of female household respondents, they were queried regarding their previous pregnancies through the preceding birth technique (within a recall period of 5 years) to estimate perinatal mortality, neonatal mortality, and infant mortality. Questions related to women’s health seeking behavior during pregnancy, delivery, and after delivery were also asked in relation to their last pregnancy (within a recall period of 2 years). Both men and women were asked questions about socio-economic status, survival or deaths of their adult sisters and births and deaths in the household over the preceding year, in order to estimate maternal mortality, under-five mortality, and crude (household) mortality rates of the previous year. Interviewers administered the questionnaires only after obtaining written informed consent from the interviewee. All questionnaires were anonymous.

In accordance with the study objectives of estimating maternal and perinatal mortality specifically in Lagos, data were collected only on births and deaths that took place in Lagos. Thus, to avoid confounding factors of differing health systems, policies, and contexts beyond Lagos, births and deaths that occurred outside of Lagos, whether in another state of Nigeria or in another country such as Benin Republic or Togo, were excluded from the analysis.

All questionnaires were pre-tested, piloted in the field, translated, and back-translated into the most common local languages in the study communities (Egun, Pidgin, and Yoruba). A data management strategy and field manual were developed to ensure clear and appropriate procedures were followed, including quality control. The quantitative data from household survey forms were double entered into EpiData version 3.1 statistical package (Lauritsen JM. (Ed.) EpiData Data Entry, Data Management and basic Statistical Analysis System. Odense Denmark, EpiData Association, 2000–2008). Completed data were initially exported to SPSS version 16 for first level data cleaning before being subsequently exported to STATA version 13 (StataCorp. 2013. Stata Statistical Software: Release 13. College Station, TX: StataCorp LP) for analysis. Results were reported as proportions for the descriptive, univariate analysis and chi-square test was used to determine whether there is an association (or relationship) between two categorical variables. Preference for the place of delivery has been modeled in a logistic regression with ethnic group, neighborhood of residence, illiteracy and working as a sex worker to assess any possible correlation: in this case we included the predictor in the model if the test had a p value ≤0.3. With the same approach we also investigated any possible association with PNM. All the hypotheses tested were 2-tailed, and we considered statistical significance only in the presence of p values <0.05. The calculation of the maternal mortality ratio was based on the sisterhood method expounded by Graham et al. [21]:MMR = 100,000 x (1- [1- total lifetime risk](1/Total Fertility Rate)).

In our study, early neonatal death was defined as: death of a liveborn infant occurring fewer than 7 completed days from the time of birth out of the total live births; late neonatal death as: death of a liveborn infant occurring after 7 completed days of age but before 28 completed days out of the total live births; infant mortality: the number of deaths of children under one year of age out of the total live births; perinatal mortality rate: any death from the 22^nd^ week of gestation up to the first week of life out of the total live births. We expressed these indicators as the number of such deaths per 1000 live births

## Results

Nearly 4,000 inhabitants were interviewed: 3,365 (84.9%) women and 598 men (15.1%) between 15 and 49 years old with a median age of 28 years old (25^th^ percentile 25 years, 75^th^ percentile 32 years). More than half of those interviewed (55.9%) were from the Egun ethnic group. The next most commonly represented groups were Yoruba (21.9%), Ibo (13.8%), Hausa (1.2%) and others (6.8%). The ethnic groups were not homogeneously distributed among the two neighborhoods: Egun (73.2%) was the majority in Riverine and Yoruba (37.6%) and Ibo (36.6%) in Badia. Gender was equally represented among the ethnic groups.

From our sample, the primary source of income was informal sector work. Amongst female respondents, the most common source of income was trading or hawking (46.1%) followed by fishing (13.8%) and food vending (9.9%). In Badia, 31.9% (266) of all female respondents admitted working as sex workers, making this the second most common source of income after trading or hawking (36.0%) in this neighborhood. Among men, “other” was the most frequently mentioned category for their primary source of income, followed by fishing (27%) and trading/hawking (15%).

Nearly half of the sample (1,895, 47.9%) never attended school; however, the data is not homogenous within the ethnic groups or by gender. Illiteracy was quite common among the Egun (77.4%) and Hausa (46.9%) ethnic groups, and less so among the Yoruba (10.0%) and Ibo (6.0%). Men were more educated than women. More than half of the women were not literate (50.6%) compared with one-third of the men (33.3%, p<0.01). Among those who were literate, 6.8% of the men had attained a university degree compared with only 1.1% of the women (p<0.01).

Regarding sexual and reproductive health, 392 women, or 19.9%, were pregnant at the time of the survey and 63.3% (1602) of the women interviewed in Riverine, 43.8% (365) of the women interviewed in Badia, and 24% of the sex workers had given birth in the last 2 years. Antenatal care coverage (ANC) was high (96.1% in Riverine and 88.8% in Badia) during the last pregnancy. The most common place where women attended ANC were private health facilities (49%), the MSF-supported Health Centre of Aiyetoro (17%), MSF mobile clinic (13%) and TBAs (13%).

In terms of care during childbirth, 64% of female respondents reported delivering in a health facility, whether private (50.2%), MSF supported (8.1%), governmental (4.8%) or faith based (0.9%). However, over one third of the women (37.8%) delivered outside of a health facility, without access to a skilled birth attendant. This percentage is higher in Badia (65.0%) and does not change by considering only the sex workers. Figures varied greatly from one community to another (Table 1): in Makoko Riverine, private health facilities were the most common place of delivery, with over half of women (57.1%) giving birth there. In Badia East, by contrast, the largest proportion of women (37.5%) delivered with a TBA, followed by private health facility (20.3%) and home delivery (18.7%). 84.8% of women claimed they have paid something for the delivery and 94.3% declared themselves satisfied with the service received.

**Table 1:** Place of delivery, stratified by neighborhood.

	Riverine, n = 1602	Badia, n = 365	Total	P value	Paid, n = 1967
	# (%)	# (%)	# (%)		# (%)
*At home*	125 (7.8)	68 (18.7)	193 (9.8)	<0.01	115 (59.6)
*TBA*	252 (15.7)	137 (37.5)	389 (19.8)	<0.01	369 (94.6)
*Private health facility*	914 (57.1)	74 (20.3)	988 (50.2)	<0.01	980 (99.2)
*Government facility*	66 (4.1)	28 (7.7)	94 (4.8)	<0.01	85 (90.4)
*Neighbourhood nurse*	75 (4.7)	29 (7.9)	104 (5.3)	<0.05	102 (97.1)
*Faith based facility*	11 (0.7)	7 (1.9)	18 (0.9)	<0.05	15 (83.3)
*MSF health facility*	143 (8.9)	16 (4.4)	159 (8.1)	<0.01	0 (0)
*Others*	13 (0.8)	2 (0.5)	22 (0.5)		3 (13.6)
Not indicated	3 (0.2)	4 (1.1)	7 (0.3)		
**Total deliveries in health facilities**	**1134 (70.7)**	**125 (34.2)**	**1259 (64.0)**	**<0.01**	

To better understand the preference of the women, we aggregated the choices in two main groups: delivery in health facility (government, private, MSF supported, faith based) and at home (TBA, at home, neighborhood nurse) and, using logistic regression, we modeled the four main variables associated with preferred place of delivery (neighborhood, ethnic group, illiteracy and years living in Lagos) (Table 2).

**Table 2:** Factors associated with delivery in health facility through multivariate analysis.

	Before adjustment			After adjustment	
		OR (95% Cl)	p value	OR (95% Cl)	p value
*Living in Riverine*		4.71 (3.7–5.9)	<0.01	2.21 (1.8–3.0)	<0.01
*Ethnic group*	Yoruba	6.48 (0.7–58.0)	0.1	5.71 (0.6–53.0)	0.1
	Hausa	10.85 (1.0–114.6)	0.47	7.33 (0.6–81.8)	0.1
	Ibo	4.11 (0.4–37.3)	0.2	2.98 (0.3–27.9)	0.3
	Egun	1.11 (0.12–10.0)	0.9	1.41 (0.1–13.1)	0.7
	Others	3.84 (0.4–35.6)	0.2	3.02 (0.3–28.7)	0.3
*Working as SW*		0.29 (0.1–0.5)	<0.01	0.97 (0.5–1.7)	0.9
*Literate*		0.33 (0.2–0.4)	<0.01	0.88 (0.6–1.1)	0.4

After covariate adjustment, only living in Makoko Riverine seems to have a significant influence on the choice of place of delivery.

When explicitly asked what factors influenced the place of their last delivery, women interviewees were able to give more than one reason, with the most common response being proximity to home (32.4%) (Table 3). Other commonly cited reasons included trust/familiarity with the system (16.1%), husband/family decision (11.7%), and previous experience delivering in the same location (10.5%).

**Table 3:** Factors influencing place of delivery as reported by the mothers.

Reason	Riverine (n = 1602)	%	Badia (n = 365)	%	Total	%	p value
*Proximity to home*	644	40.2%	133	36.4%	777	39.5%	0.2
*Trust /Familiarity in system*	304	19.0%	82	22.5%	386	19.6%	0.1
*My husband/family’s decision*	250	15.6%	30	8.2%	280	14.2%	<0.01
*I have already delivered there in the past*	235	14.7%	18	4.9%	253	12.9%	<0.01
*Affordability / Cost*	71	4.4%	32	8.8%	103	5.2%	<0.01
*They have referred me there*	87	5.4%	16	4.4%	103	5.2%	0.4
*Quality of service*	79	4.9%	9	2.5%	88	4.5%	0.03
*There is not discrimination*	72	4.5%	6	1.6%	78	4.0%	0.02
*Availability of drugs*	40	2.5%	13	3.6%	53	2.7%	0.2
*Availability of Qualified medical personnel*	43	2.7%	5	1.4%	48	2.4%	0.1
*Cultural reasons*	22	1.4%	4	1.1%	26	1.3%	0.6
*MSF referral*	16	1.0%	6	1.6%	22	1.1%	0.3
*Others*	119	7.4%	65	17.8%	184	9.4%	<0.01

Of the 1967 pregnancies reported by the female respondents (when asked about their last pregnancy in the last 2 years), 140 ended with the death of the child (7.1%). The timing of neonatal and infant deaths related to the last pregnancy is shown in Table 4 and compared with the outcomes of the last 5 years (6897 pregnancies, including the pregnancies of the last 2 years) to appreciate the trend.

**Table 4:** Timing of neonatal and infant deaths.

	At the end of the last pregnancy (n = 1967)		Over a 5 years period* (n = 6897)	
Indicator	#	‰	#	‰
*Perinatal mortality*	67	34.5	249	36.6
*Early neonatal mortality*	40	20.6	146	21.5
*Late neonatal mortality*	15	7.7	15	2.2
*Neonatal mortaliy*	55	28.4	161	23.7
*Infant mortality*	85	43.8		

* Induced abortions (500) have been excluded

We estimated a PNM of 34.5 deaths for 1,000 live births in relation to the last pregnancy and a PNM of 36.6 deaths for 1,000 live births by considering the last 5 years. To better understand the magnitude of child mortality, on the basis of the data collected related to the last pregnancy (2 years recall period), we stratified the main indicators by place of delivery (health facility or home), dwelling place (Riverine and Badia), most prevalent ethnic group (Yoruba and Egun) and by the profession of sex worker. Those residing in Badia and working as a sex worker in Badia had significantly worse indicators than those residing in Riverine and those earning a living through other female professions (Table 5).

**Table 5:** Child mortality indicators stratified by place of delivery, dwelling place, ethnic group and by the profession of sex worker.

Indicator	Health Facilities (n = 1259)			at Home (n = 686)			Riverine (n = 1602)			Badia (n = 365)			Yoruba (n = 392)			Egun (n = 1220)			SW (n = 64)		
* *	#	‰		#	‰	p	#	‰		#	‰	p	#	‰		#	‰	p	#	‰	p*
*Early neonatal mortality*	24	19.1		16	23.6	0.5	29	18.3		11	30.9	0.1	7	18.0		27	22.4	0.6	3	48.4	0.1
*Late neonatal mortalty*	7	5.6		8	11.8	0.1	7	4.4		8	22.5	**<0.01**	3	7.7		5	4.1	0.4	3	48.4	**<0.01**
*Neonatal mortality*	31	24.7		24	35.5	0.2	36	22.7		19	53.4	**<0.01**	10	25.7		32	26.6	0.9	6	96.8	**<0.01**
*Perinatal mortality*	40	31.8		25	36.9	0.5	47	29.7		20	56.2	**<0.05**	10	25.7		42	34.9	0.3	5	80.6	**<0.05**
*Infant mortality*	15	11.9		10	14.8	0.6	16	10.1		10	28.1	**<0.01**	7	18.0		12	10.0	0.2	4	64.5	0.4

* Values compared with the rest of the population not working as SW

Finally, we further investigated possible associations with PNM through multivariate analysis (logistic regression) by considering in the model place of delivery, neighborhood, belonging to the Egun ethnic group, illiteracy and working as a sex worker (Table 6).

**Table 6:** Factors associated with PNM through multivariate analysis.

	Before adjustment		After adjustment	
	OR (95% Cl)	p value	OR (95% Cl)	p value
*Living in Riverine*	0.77 (0.4–1.3)	0.3	0.29 (0.1–0.7)	<0.01
*Delivery in health facility*	0.71 (0.5–0,9)	<0.05	0.71 (0.5–0.9)	<0.05
*Being Egun*	1.33 (0.8–2.2)	0.3	2.5 (1.1–5.7)	<0.05
*Working as SW*	0.93 (0.4–2.3)	0.9	1.1 (0.1–10.4)	0.9
*literate*	1.0 (0.8–1.2)	0.9	1.6 (0.2–14.9)	0.6

After adjustment, only three variables were statistically associated with the outcome of death from the 22^nd^ week of gestation up to the first week of life: living in Riverine and delivering in a health facility as protective factors and belonging to the Egun ethnic group as a risk factor.

We calculated the under-five mortality rate (U5MR) using as the denominator the number of pregnancy outcomes accounted for in the last five years resulting in a U5MR of 103 per 1,000.

To estimate the primary outcome of this study, maternal mortality, using the sisterhood method, we estimated the life-time risk of maternal death in these communities to be 0.0556 or 1 in 18.

With a TFR of 5.4, this translates into an estimated maternal mortality ratio of 1050 per 100,000 with a 95% confidence interval of 894 to 1215.

Of the total 5,430 pregnancies reported in the last 5 years, 500 induced abortions (9.2%) were self-reported by female study respondents.

## Discussion

This is a first-time application by MSF-OCBA (in partnership with local government and academic/medical institutions) of a widely-accepted, feasible, and cost-effective method for estimating maternal mortality: the sisterhood method. We carried out the study with the aim of investigating possible inequalities among the most neglected groups and the rest of the population with regards to maternal and child health.

The maternal mortality ratio of 1,050 per 100,000 live births estimated for the two urban slum communities of Lagos targeted by this survey is very high. It is nearly double the figure previously estimated for Lagos State of 545 per 100,000 live births [3] and is also considerably higher than the national figure of 814 [20]. The lifetime risk of maternal death (1 in 18) in these two communities is also more than double the estimate of 1 in 42 for Lagos State [3] and is also higher than the previous national estimate of 1 in 23 [21]. Thus, the maternal mortality findings of this study highlight the disparities among health and survival status amongst poor, ostracized, and often neglected groups living within Lagos city. Discrepancies between the most neglected and wealthy areas were underlined also by another study, carried out in Nairobi City from 2003 to 2005. In this case, information about maternal death were gathered through verbal autopsy interviews and a MMR of 706/100,000 live births was estimated in the slum population (in 2005 the national MMR in Kenya was estimated at 560/100,000 live births) [22].

With regards to neonatal, infant, and under-five mortality rates, this study estimates a 28 per 1,000, 44 per 1,000, and 103 per 1,000 (respectively) and a perinatal mortality rate of 34.5 consistent with estimates for the South-West region (36 per 1,000) [23]. Women living in Badia and working as sex workers suffered the worst PNM (56.2 and 80.6 respectively) and, based on multivariate analysis, living in Badia (p <0.01), belonging to Egun ethnic group (p <0.05) and delivery outside a health facility (p <0.05) are correlated to high PNM. Proximity to home is reported as the main reason for the choice of delivery both in Riverine and Badia. Also in Riverine the husband/family decision played a more important role than in Badia (15.6% vs 8.2%, p <0.01) indicating perhaps a much stronger and more influential family and social system. In Badia, affordability is more of an issue compared to Riverine (8.8% vs 4.4%, p <0.01).

Limitations of this study include the following: the sisterhood method relies on a verbal autopsy approach and generates a retrospective estimate of maternal mortality (5 years in our study). Thus, a certain level of recall bias common in many observational studies must be taken into account. Sensitive subjects such as the death of a child and abortion could be underreported due to, for example, cultural beliefs surrounding talking about a death that happened in the household and the fear that doing so could invite more negative events. Finally, the findings of this study cannot be extrapolated to the whole city of Lagos (or to other underserved, urban slums with marginalized populations) but may only be relevant to the two particular neighbourhoods that were the subject of this study.

Strengths of this study include generating robust, essential, sub-national level data for program & policy planning from a marginalized urban slum area/population, where MMR had never before been estimated and where routine, reliable health data (e.g., civil registration and vital statistics system) was generally not available. The study, therefore, makes an important contribution to the current literature, which generally reflects a dearth of sub-national level maternal mortality data, especially among marginalized and vulnerable populations in countries suffering the highest levels of maternal deaths, such as Nigeria. The “health equity gap” within countries can be significant and severe, resulting in far worse survival and health outcomes for women, newborns and children who are poor, underserved, ostracized, or hard-to-reach [24].

The methodology used in this study could potentially be applied in other settings, especially where national level maternal mortality estimates mask inequalities and disparities within a country, to help countries obtain maternal mortality estimates from deprived, neglected areas. This methodology may also be employed as an interim solution while a country is in the process of developing its capacity to count every maternal death through, for example, a well-established maternal death surveillance and response system, implemented at scale.

By ensuring inclusion of vulnerable/marginalized sub-populations including sex workers and ethnic minorities, our research aims to provide a comprehensive picture of the health conditions of mother and child. To pursue this scope we adjusted the original questions proposed by the sisterhood methodology to exclude births delivered and sisters living outside the study area (the sisterhood method generally does not inquire about the location of sisters’ residence or the place of deaths). This adjustment of the original questionnaire elaborated by Graham and standardized by the WHO allows us to better explore MMR at subnational level and to target specific vulnerable groups.

The findings of this study–in particular, the very high level of maternal mortality–should serve as a clarion call to action and advocacy to improve maternal health and survival amongst the most vulnerable and marginalized groups within urban Lagos. To remedy such disparities and truly achieve the vision enshrined in the Sustainable Development Goals of “leaving no one behind”, targeted, equity-focused policies and programmes for neglected and marginalized subgroups of the society are critically needed, as inequalities often exist and may be severe, yet are often masked by national level indicators.

Specific, targeted, timely, and evidence-based actions must be planned, implemented, monitored, and evaluated to improve maternal health among the most vulnerable, and to ensure the elimination of the inequalities highlighted in this study. All key stakeholders concerned with health, human rights, and development in Nigeria should work together to respond to this call and tackle the root causes of the problem. For example, the finding that over one-third of female respondents had delivered outside of a health facility, underscores the need to promote increased access to and utilization of skilled birth attendance, with specific attention to providing equitable access to all members of society (without discrimination). Existing services, programs and policies must ensure a client-centred, culturally sensitive approach. This includes being accessible, acceptable, affordable and welcoming to marginalized groups such as the Egun people or other ethnic minorities who may not speak the “mainstream” languages, as well as to sex workers, who may feel shunned or discriminated against by society and community generally, and may fear the way that health workers might treat them if they were to seek antenatal or delivery care in a health facility. By employing such equity-focused approaches and ensuring that specific strategies are implemented to gather data and monitor inequalities among even the most vulnerable and excluded populations, Nigeria will increase its chances of reaching the Sustainable Development Goals, ending preventable maternal and child deaths, and leaving no woman or child behind.
